# Insights into the Cross-Population Transferability of Polygenic Scores for Substance Use

**DOI:** 10.1007/s10519-026-10265-1

**Published:** 2026-04-29

**Authors:** Gretchen R. B. Saunders, Scott Vrieze, Dajiang J. Liu

**Affiliations:** 1 Department of Psychology, University of Minnesota, Minneapolis, MN USA; 2 Department of Public Health Sciences, Penn State College of Medicine, Hershey, PA USA

**Keywords:** Substance use, Tobacco, Alcohol, Polygenic score, Genetic ancestry

## Abstract

Polygenic scores have potential for predicting health outcomes based on genomic data. However, their portability across diverse populations is hindered by the overrepresentation of European ancestries in discovery genome-wide association (GWAS) studies. The current study evaluated the transferability of substance use polygenic scores across European, African, American, and East Asian populations. European-based scores demonstrated reduced predictive accuracy in non-European populations with mean relative accuracies of 36% in American, 22.5% in East Asian, and 3.8% in African ancestries. Beyond discrete genetic ancestry groups, we found that European-based score prediction declined as a function of genetic distance from the European-stratified GWAS. That is, predictive accuracy declines as genetic distance, derived from continuous measures of ancestry, from the discovery GWAS increases. We evaluated the extent to which differences across populations in linkage disequilibrium (LD), allele frequency, and heritability explained the reduction in accuracy. Across phenotypes we found that approximately 56.0% of accuracy decline in American ancestries was explained by LD and allele frequency differences, with differences in LD appearing to be the primary factor. For East Asian ancestries this was 67.1% and was 84.0% in African ancestries. Differences across populations in heritability accounted for only small portions of reduced polygenic score transferability. This suggests that LD and frequency differences explain significant portions of accuracy loss, but substantial reductions persist, suggesting contributions from other factors like causal effect size differences. The current study provides important information on the causes and extent of the polygenic score transferability problem and has important implications for refinement of polygenic scores. Expanding the diversity of GWAS and target samples remains essential for improving accuracy and equity in prediction.

**Supplementary Information:**

The online version contains supplementary material available at 10.1007/s10519-026-10265-1.

## Introduction

Polygenic risk scores (PRS) derived from genome-wide association studies (GWAS) are frequently used to predict an outcome of interest at the individual level. Scores have been developed to predict disease, disorder, anthropometric measures, social outcomes, and behaviors. As GWAS sample sizes have increased over time, the precision of PRS and corresponding predictive accuracy have improved. This has led to increased optimism for the clinical utility of polygenic scores (Lambert et al. 2019; Torkamani et al. 2018).

One major impediment to this promise, however, is the overrepresentation of individuals of inferred European (EUR) ancestry in GWAS discovery samples. It has become apparent that the predictive accuracy of PRS is maximized in samples in which the genetic ancestry matches that of the discovery GWAS sample (Duncan et al. 2019; Martin et al. 2019; Mostafavi et al. 2020). Predictive accuracy declines as a function of the genetic differences, or distance, between discovery and target samples (Ding et al. 2023; Kamiza et al. 2022). Because of this, scores developed from current GWAS, the majority of which are based on individuals of European ancestries, are less likely to be useful, clinically or otherwise, in individuals of different inferred ancestries. The greater the difference, the less predictive will be the score.

Recent work has begun to identify the factors that lead to this lack of PRS transferability. These factors may include differences across populations in linkage disequilibrium (LD) patterns and allele frequencies, differences in causal variant effect sizes, due to gene-environment or gene-gene interactions, amongst other possibilities (Kachuri et al. 2024; Novembre and Barton 2018). GWAS identified SNPs are themselves unlikely to be causal but rather are in LD with a nearby causal variant. Thus, if LD between a PRS SNP and causal variant differs between two populations, the PRS based on that SNP will explain more of the phenotypic variance in the population with higher LD. Additionally, because GWAS have the greatest power to detect common variants, it is likely that GWAS will tend to identify variants that are more common in the discovery sample ancestry compared to other ancestries, leading to systematically lower predictive accuracy in non-European ancestries. Importantly, both results hold even if the causal variants and their effect size are the same across populations.

Prior simulations, theoretical derivations, and empirical work have suggested that differences in LD and minor allele frequencies (MAF) contribute substantially to the observed reduction in PRS accuracy across populations (Bitarello and Mathieson 2020; Wang et al. 2020). These patterns have held for traits like height, body mass index, and several health-related diseases. The contribution of LD and MAF differences to reduced accuracy, however, is larger in some ancestries than others. In one recent study, approximately 86%, 57%, and 37% of the accuracy decline of PRS was explained by differences across populations in LD and MAF for African (AFR), East Asian (EAS) and South Asian ancestries, respectively(Wang et al. 2020). Taken together, across physical and medical outcomes, and across diverse ancestries, differences in LD and MAF account for substantial, but not all, of the decrease in predictive accuracy of PRS. Any remaining decreases in predictive accuracy appear due to other factors, such as different causal effect sizes or genetic/environmental architectures.

Previous research has several limitations that we aim to address here. First, few discovery GWAS are based on non-European ancestries [see: https://hugofitipaldi.shinyapps.io/gwas_results/ using data from the NHGRI-EBI GWAS catalog (Sollis et al. 2023)]. It is unclear what the patterns of PRS transferability looks like from AFR-, American- (AMR), and EAS- based PRS to other populations. Second, much of the existing work focuses on model phenotypes (e.g., height or simulated phenotypes) or medical/disease related outcomes. Little work has been done evaluating whether behavioral traits, that might be affected to a greater extent by environmental or cultural factors, follow similar patterns of reduced transferability. Finally, much of the work on PRS transferability makes group-level comparisons which are based, in part, on subjective thresholds. That is, genetic ancestry is frequently best modeled as a continuum (versus discrete groups) in any given sample, so the assignment of an individual to an ancestry group is routinely arbitrary. Categorizing genetic ancestry often removes individuals of recently admixed ancestries and so the results of many analyses reflect only a subset of individuals. The current study aims to address these gaps through:

1. Inclusion of greater diversity in genetic ancestries in both the discovery GWAS and target PRS samples.
2. Evaluation of the transferability of PRS for substance use traits, to compare to existing results of anthropometric and health-related outcomes.
3. Description of the transferability of PRS across inferred ancestries represented categorically and continuously.
4. Evaluation of the extent to which differences across populations in LD, MAF, and SNP heritabilities explain the reduced observed accuracy of PRS using a recently derived theoretical model as well as empirical observations.

## Methods

### Data Preparation and Quality Control

Throughout this manuscript, the terms ‘European (EUR) ancestry’, ‘African (AFR) ancestry’, ‘East Asian (EAS) ancestry’, and ‘American (AMR) ancestry’ are used as abbreviated descriptors to refer to individuals categorized into broad continental genetic ancestry clusters based on their genomic proximity to reference populations (e.g., 1000 Genomes Project). These terms serve as a shorthand for ‘individuals of predominantly [European/African/etc.] inferred genetic ancestry’ to enhance the readability and conciseness of the text. We acknowledge that these labels do not capture the full complexity or the fine-scale ancestral diversity within these groups.

*Discovery GWAS*. Genome-wide association study (GWAS) summary statistics were taken from Saunders et al. (2022) which included the phenotypes of smoking initiation (SmkInit; whether or not an individual had ever smoked regularly; higher score = has regularly smoked), age of initiation of regular smoking (AgeSmk), cigarettes per day among those who ever smoked regularly (CigDay), smoking cessation (SmkCes; whether or not an individual reports being a current of former smoker; higher score = current smoking), and alcoholic drinks per week (DrnkWk). The discovery GWAS conducted both ancestry stratified and multi-ancestry meta-analyses. We make use of the ancestry-stratified meta-analytic results here for four major ancestry populations: European ancestry, African ancestry, American ancestry, and East Asian ancestry. Discovery GWAS samples sizes varied across phenotype and ancestry with a minimum *N* = 17,508 for AFR stratified AgeSmk results and a maximum of *N* = 2,669,029 for EUR stratified SmkInit results.

*Target Sample.* The *All of Us* Research Program (AoU) was used as a validation sample for all analyses. The *All of Us* Research Program (Bick et al. 2024) is an initiative run by the National Institutes of Health aimed at collecting genetic and health data from one million adult participants in the United States. We used information from all participants who had version 7 genotype array data and had completed the lifestyle questionnaire assessing tobacco and alcohol use resulting in an initial sample size of *N* = 245,271. We used AoU’s categorical genetic ancestry predictions to further subset the sample to individuals of AFR, AMR, EAS, and EUR ancestries. Finally, we removed one individual at random within a pair if the kinship coefficient was greater than 0.1. This results in a final sample size of *N* = 217,812 (*N* = 48,616 AFR; *N* = 39,491 AMR; *N* = 5,367 EAS; *N* = 124,338 EUR). Descriptive statistics of both the discovery GWAS and the target sample (AoU) are given in Table 1.

**Table 1:** Descriptives statistics of datasets used in the analysis

Target sample	Ancestry	% Female	Age M (SD)	*SmkInit* %	*AgeSmk* M (SD)	*CigDay* M (SD)	*SmkCes* %	*DrnkWk* M (SD)
All of Us	EUR	59.4	55.9 (17.0)	42.7 *N* = 122,656	17.8 (6.1) *N* = 48.399	16.1 (11.9) *N* = 44,631	71.6 *N* = 51,831	4.2 (6.9) *N* = 96,345
	AFR	56.6	49.4 (14.8)	48.2 *N* = 47,743	18.9 (7.6) *N* = 19,181	11.1 (9.8) *N* = 17,672	27.3 *N* = 22,591	4.0 (8.5) *N* = 29,948
	AMR	66.0	44.8 (15.7)	29.8 *N* = 38,934	18.1 (6.5) *N* = 10,085	10.5 (10.4) *N* = 9,153	52.7 *N* = 11,403	3.0 (7.4) *N* = 23,396
	EAS	63.4	44.4 (17.2)	17.6 *N* = 5,298	-	-	-	2.1 (4.1) *N* = 3,674

Discovery GWAS	Ancestry	% Female	Age M (SD)	SmkInit N	AgeSmk N	CigDay N	SmkCes N	DrnkWk N
	EUR	-	-	2,669,029	618,541	618,489	1,147,272	2,428,851
	AFR	-	-	119,589	17,508	20,157	34,970	95,343
	AMR	-	-	286,026	33,914	35,129	90,525	274,707
	EAS	-	-	296,395	-	-	-	160,775

All of Us was used as the independent target sample. Ancestry-stratified GWAS summary statistics were taken from Saunders et al. (2022)

M = mean; SD = standard deviation; N = sample size; EUR = European; AFR = African; AMR = American; EAS = East Asian; sex and age distribution information was not available for the discovery GWAS sample. EAS stratified analyses for AgeSmk, CigDay, and SmkCes were not conducted due to low validation sample sizes (Ns < 1,000)

### Polygenic Score Generation

For each set of summary statistics we used PLINK (Chang et al. 2015) to clump SNPs based on LD structure. AoU ancestry-stratified genotypes were used as the reference with ancestry matched to that of the discovery GWAS. For individuals in AoU of EUR, AFR, and AMR ancestry, we randomly selected 10k genotypes to serve as the LD reference. We used all individuals of EAS ancestry for LD estimation, as the total EAS sample size was less than 10,000. A sequence of *p*-value, LD, and physical distance thresholds were used: *p*-value thresholds for index SNPs of 5e-5, 0.01, 0.5; LD r^2^ thresholds of 0.1 and 0.5; distance thresholds of 100 kb and 250 kb. This resulted in 12 scores per discovery ancestry and phenotype combination.

Polygenic scores were then generated in PLINK by adding up the product of minor allele counts multiplied by the effect sizes from the respective GWAS summary statistics:

$$\:{PRS}_{i}=\:\sum\:_{i=1}^{M}{\beta\:}_{j}{G}_{ij}$$

Where $\:{PRS}_{i}$, the polygenic score for individual $\:i$, is the product of effect sizes $\:{\beta\:}_{j}$ for variant $\:j$ and $\:{G}_{ij}$, the allele dosage of individual $\:i$ at variant $\:j$, summed over $\:M$ PRS SNPs.

### Polygenic Score Predictive Accuracy

Prediction accuracy was estimated based on the regression of a given phenotype (SmkInit, AgeSmk, CigDay, SmkCes, and DrnkWk) on the polygenic score along with a set of standard covariates, which included age, age^2^, sex, the interaction between age and sex, and the first ten genetic principal components. We first performed this regression without the PRS. Then, the PRS predictor was added to the regression model and the difference in R^2^ was calculated. For our quantitative phenotypes, AgeSmk, CigDay, and DrnkWk, the predictive power of the PRS was the change in the R^2^ between the regression without the PRS and the regression with the PRS. For our two binary phenotypes, SmkInit and SmkCes, we calculated the change in R^2^ on the liability scale (Lee et al. 2012) from logistic regressions. Confidence intervals around all incremental R^2^ values were bootstrapped with 1,000 replications. We report 95% percentile-based confidence intervals.

### Genetic Distance

To estimate PRS accuracy across individuals while avoiding the potential arbitrariness with which ancestry groups are assigned, we defined the genetic distance between the discovery GWAS sample from Saunders et al. (2022) and each individual in the All of Us target sample. To do so, the discovery GWAS multi-dimensional scaling (MDS) components based on the per ancestry allele frequencies were projected onto the principal component (PC) spaces of AoU. This involved several steps detailed below.

First, we projected AoU PCs onto those of 1000 Genomes (1000 Genomes Project Consortium et al. 2015) using an online bias-adjusted projection (OADP) method implemented in FRAPOSA (Zhang et al. 2020) (Supplementary Fig. 1). This method corrects shrinkage bias in PC projection caused by the number of variants far exceeding the number of individuals in the reference sample (Dey and Lee 2019), here 1000 Genomes (1000 Genomes Project Consortium et al. 2015).

Next, we extracted ancestry-stratified allele frequencies from 1000 Genomes and the discovery GWAS for variants that existed in all sources (i.e., variants common to all ancestry-stratified 1000 Genomes and GWAS summary statistics files). Multidimensional scaling (MDS) components of the allele frequency matrix were computed based on Euclidian distance. In this way we plotted the similarities (e.g., the similarity between the GWAS EUR data and 1000 Genomes AFR data) across the discovery GWAS and 1000 Genomes.

**Fig. 1:**
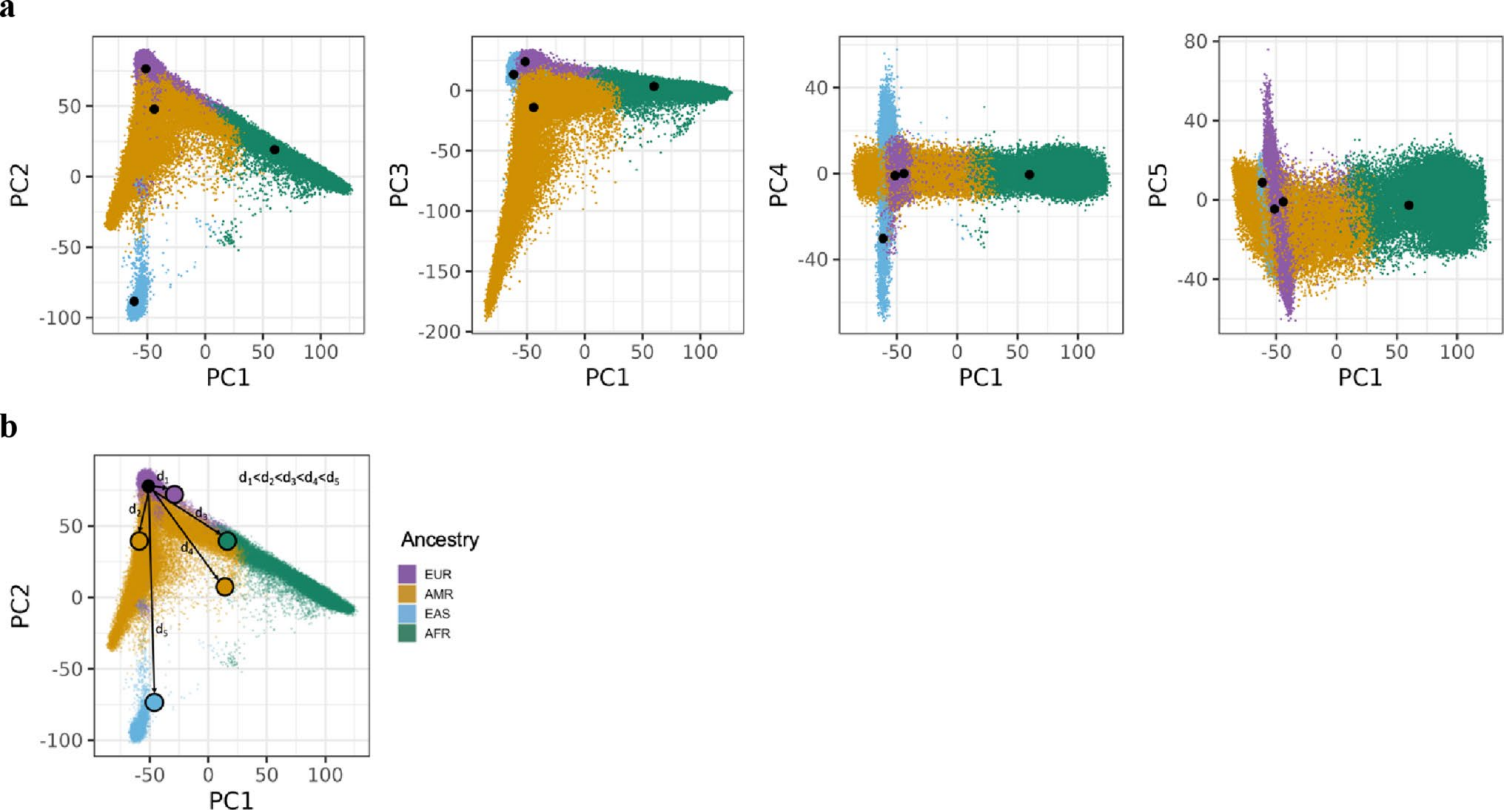
Mapping of ancestry-stratified GWAS discovery samples onto principal components (PC) space of the All of Us validation sample and illustration of genetic distance metric. **a** Results of the Procrustes transformation applied to the GWAS discovery sample MDS points (black circles) onto the PC space of the All of Us sample (colored circles) for the first five PCs. This is the PC space based on All of Us individuals that also contains GSCAN per-ancestry MDS components while retaining the relative similarities. **b** Example illustration of how the genetic distance from a given GWAS discovery sample is defined for individuals in All of Us, adapted from Ding et al. (2023). Genetic distances are defined as the Euclidean distance between an individual’s PC point and a given MDS point across the first five dimensions. Solid black point denotes the EUR-stratified discovery GWAS sample. Large colored points in black outline denote five example individuals from All of Us, colored by their assigned global ancestry, with length of arrows indicating the Euclidean distance from the discovery GWAS

Using the first five MDS components for 1000 Genomes and the corresponding PC centroids, we used a Procrustes transformation to map the MDS components onto the combined PC space (Supplementary Fig. 2). This uses the per ancestry PC centroids for 1000 Genomes as a target matrix and modifies the full MDS matrix, including the discovery GWAS MDS components, based on a scaling factor, translation vector, and rotation matrix, to place the MDS matrix in the space of the PCs (Fig. 1a). The result is a PC space based on AoU individuals that also contains the discovery GWAS per-ancestry MDS components while retaining the relative similarities.

From here we define genetic distance between each individual in the target samples and the discovery GWAS sample simply by the Euclidian distance between their PC point and the GWAS MDS point across the first five dimensions. (Fig. 1b).

### Theoretical Model of the Relative Accuracy of PRS

We used a recently derived theoretical model (Wang et al. 2020) that estimates the relative accuracy of PRS between two populations with the following equation:

$$\begin{array}{l}RA = \frac{{R_{pop2}^2}}{{R_{pop1}^2}} \approx \frac{{\rho _b^2h_{pop2}^2}}{{h_{pop1}^2}} \times {\left( {\frac{{\mathop \sum \nolimits_{k = 1}^M \overline {{r_{k,~pop1}}{r_{k,pop2}}} \sqrt {\frac{{{p_{k,pop2}}\left( {1 - {p_{k,pop2}}} \right)}}{{{p_{k,pop1}}\left( {1 - {p_{k,pop1}}} \right)}}} }}{{\mathop \sum \nolimits_{k = 1}^M \overline {r_{k,pop1}^2} }}} \right)^2}\\\quad \quad \times \frac{{\mathop \sum \nolimits_{k = 1}^M {p_{k,pop1}}\left( {1 - {p_{k,pop1}}} \right)\hat \beta _k^2}}{{\mathop \sum \nolimits_{k = 1}^M {p_{k,pop2}}\left( {1 - {p_{k,pop2}}} \right)\hat \beta _k^2}}\end{array}$$

In this case Population 1 ($\:pop1$) refers to the discovery GWAS population while Population 2 ($\:pop2$) refers to the target population. So the relative accuracy (RA) between these two populations ($\:{R}_{pop2}^{2}/{R}_{pop1}^{2}$) is a function of the heritabilities of the phenotype ($\:{h}_{pop1}^{2}$ and $\:{h}_{pop2}^{2}$) in each population, the correlation of causal SNP effects ($\:{\rho\:}_{b}$) between the two populations, minor allele frequencies $\:{p}_{k,pop1}$ and $\:{p}_{k,pop2}$ at the $\:k$th SNP included in the PRS, and LD between the $\:k$th PRS SNP and the $\:j$th causal SNP. Because we do not know the true causal SNPs, we follow the same heuristic as the authors of the method that takes any SNP within a 100 kb window that is in LD ($\:{r}^{2}>0.45$) with a given PRS SNP as a candidate causal SNP. So then $\:\stackrel{-}{{r}_{k,pop1}^{2}}$ is the mean squared correlation between the $\:k$th PRS SNP and each candidate causal SNP (within 100 kb) and $\:\stackrel{-}{{r}_{k,\:pop1}{r}_{k,pop2}}$ is the mean LD between the $\:k$th PRS SNP and each candidate causal SNP in each population. As we do not know the true causal SNPs, we assume that the correlation of casual SNP effects is equal to one (i.e., $\:{\rho\:}_{b}$=1).

The RA predicted by the above equation, termed $\:{RA}_{LD+MAF+{h}^{2}}$ (under the assumption that $\:{\rho\:}_{b}$=1) can then be compared to the observed relative accuracy, $\:{RA}_{OBS}$. If $\:{RA}_{LD+MAF+{h}^{2}}>{RA}_{OBS}$, we infer that differences in LD, MAF, and/or heritability across populations drive at least some of the reduced accuracy. We follow the same comparison as Wang et al. (2020) to compute the proportion of the loss of accuracy (LOA) as $\:{LOA}_{LD+MAF+{h}^{2}}=\frac{\left(1-{RA}_{LD+MAF+{h}^{2}}\right)}{\left(1-{RA}_{OBS}\right)}\times\:100\%$. This LOA term can be interpreted as the proportion of observed reduced accuracy that can be explained by differences between populations in LD, MAF, and heritability.

Further, to isolate the specific impact of each of these factors on reduced accuracy, we evaluate three constrained versions of this model:

1. $\:{RA}_{LD+MAF}$: Computed by assuming cross-population heritability is equal ($\:{h}_{pop1}^{2}$= $\:{h}_{pop2}^{2}$) and genetic correlation is perfect ($\:{\rho\:}_{b}$=1). This captures the combined effect of LD tagging and allele frequency differences.
2. $\:{RA}_{LD}$: Calculated by isolating the mean LD and squared correlations between PRS SNPs and candidate causal variants, holding MAF and $\:{h}^{2}\:$effects constant.
3. $\:{RA}_{MAF}$: Calculated under the assumption that all PRS SNPs are themselves the causal variants, thereby removing LD tagging effects and focusing solely on the variance explained by frequency differences:

$$\begin{array}{l}R{A_{MAF}} = {\left( {\frac{{\mathop \sum \nolimits_{k = 1}^M \sqrt {\frac{{{p_{k,pop2}}\left( {1 - {p_{k,pop2}}} \right)}}{{{p_{k,pop1}}\left( {1 - {p_{k,pop1}}} \right)}}} }}{M}} \right)^2}\\\quad \quad \times \frac{{\mathop \sum \nolimits_{k = 1}^M {p_{k,pop1}}\left( {1 - {p_{k,pop1}}} \right)\hat \beta _k^2}}{{\mathop \sum \nolimits_{k = 1}^M {p_{k,pop2}}\left( {1 - {p_{k,pop2}}} \right)\hat \beta _k^2}}\end{array}$$

For each RA model, we compute respective proportion of the loss of accuracy ratios (e.g., $\:{LOA}_{MAF}=\frac{\left(1-{RA}_{MAF}\right)}{\left(1-{RA}_{OBS}\right)}\times\:100\%)$. Standards errors (SE) for each term have been derived by the original authors in their Supplementary Note. We use these same derivations to report standard errors and normal approximation 95% CIs ($\:\pm\:1.96\times\:\mathrm{S}\mathrm{E}$).

### Recombination Maps

We used recombination maps estimated in the CEU population of 1000 Genomes and in an African American sample (Hinch et al. 2011). We took a 20Kb window around each PRS SNP and used linear interpolation to estimate the per variant recombination rate. We then split the PRS SNPs for each phenotype into four equally sized bins based on recombination rate and computed the variance explained within each validation sample ancestry. We report point estimates and 95% confidence intervals from the percentile bootstrap over 1000 replicates per bin/ancestry combination.

## Results

### Polygenic Score Selection

We evaluated the predictive accuracy of the 12 PRS per discovery ancestry and phenotype combination generated above (full results for all score combinations are included in the Supplementary Table 1). Analyses for EAS ancestry individuals were not conducted for AgeSmk, CigDay, and SmkCes due to low target sample sizes (*N* = 768 to 916). Across PRS ancestries and phenotypes, we found that the predictive accuracy of scores tended to increase as the *p*-value threshold was relaxed. Within a set *p*-value threshold, higher LD r^2^ thresholds (i.e., r^2^ of 0.5) also performed better. There was little difference in predictive accuracy between the 100 kb and 250 kb window size. We note, however, that there is some variability in these patterns suggesting that there is no universal set of *p*-value and LD threshold values that maximize accuracy in all cases.

Overall, as expected given substantially larger GWAS discovery sample sizes, predictive accuracy in EUR ancestries was generally higher than for other ancestries. Variance explained by SmkInit scores were also generally higher than for other phenotypes. This was also expected as the GWAS discovery sample sizes for each ancestry were largest for SmkInit. One notable exception is the EAS-based PRS for DrnkWk in EAS individuals which greatly outperforms all other DrnkWk scores. This result is driven primarily by rs671 in the *ALDH2* gene, a known variant with a large effect on alcohol metabolism, alcohol use, and alcoholism that is common in EAS ancestries but extremely rare in other ancestries. Based on these results, we selected, for each AoU ancestry, the PRS that maximized the predictive accuracy within ancestry. For each of these selected scores, we then estimated the predictive accuracy in all individuals in AoU to evaluate transferability across populations.

### Transferability of Polygenic Scores by Genetic Ancestry

**Fig. 2:**
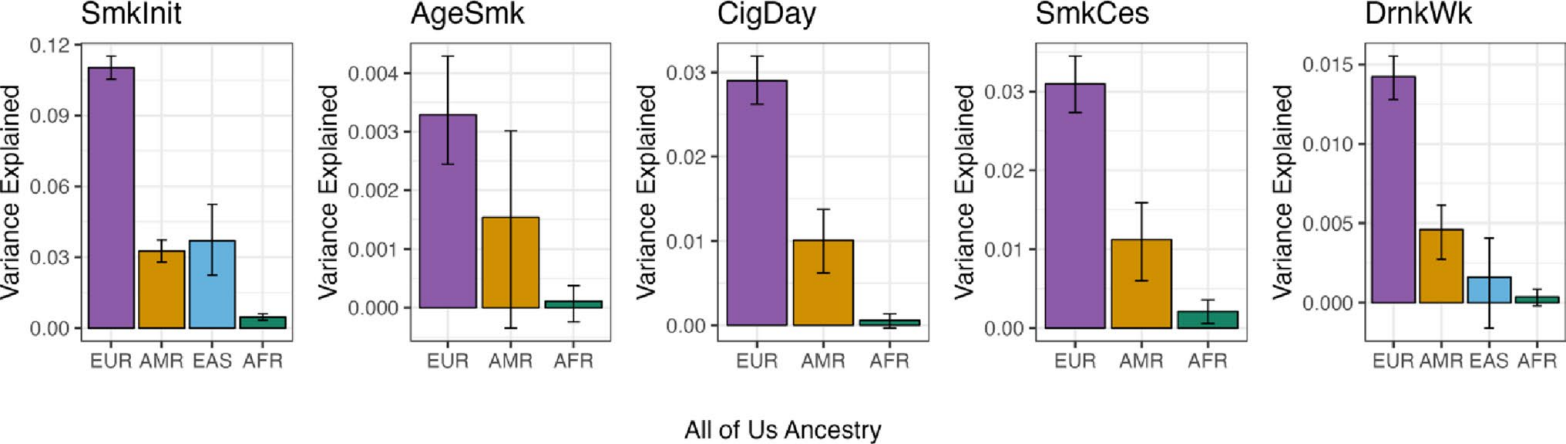
Variance explained by EUR-based polygenic scores in independent validation samples of European (EUR), American (AMR), East Asian (EAS), and African (AFR) ancestries for all five phenotypes. Individuals of EAS ancestry were removed for AgeSmk, CigDay, and SmkCes due to low validation sample sizes. Error bars denote 95% confidence intervals from percentile bootstrapping with 1000 replications

The predictive accuracy of EUR-based PRSs, for each phenotype, is shown in Fig. 2. EAS ancestry for AgeSmk, CigDay, and SmkCes were removed due to low target sample sizes. European-based scores explain the largest amount of variance for each phenotype in AoU EUR ancestry individuals (ranging from 0.32 to 11%), followed by AoU AMR ancestry individuals (0.15% to 3.3%), then EAS ancestry individuals (0.14 to 3.7%), and finally AFR ancestry individuals in AoU (0.01 to 0.47%). The mean relative accuracy of EUR-based scores (i.e., the variance explained by the score in non-EUR ancestries relative to the variance explained by the same score in EUR ancestries) was 36.0% in AMR ancestries, 22.5% in EAS ancestries, and 3.8% in AFR ancestries across phenotypes.

### Transferability of Polygenic Scores by Genetic Distance

While ancestry group-level comparisons are informative for understanding general patterns of PRS transferability, they mask within ancestry heterogeneity and rely on somewhat subjective thresholds for ancestry group assignment. To address these shortcomings, we additionally evaluated the predictive accuracy of PRS as a function of genetic ancestry distance (GD) from the GWAS discovery sample. Taking each discovery GWAS sample (e.g., EUR SmkInit GWAS) as a reference point in the MDS ancestry space, we calculated 20 equal-sized bins of genetic distance between this reference point and each individual in AoU. We then estimated the predictive accuracy of the ancestry matched PRS within each bin of genetic distance (e.g. EUR-based SmkInit PRS within each bin of genetic distance to the discovery GWAS EUR SmkInit sample). This process was repeated for each GWAS ancestry/phenotype combination.

**Fig. 3:**
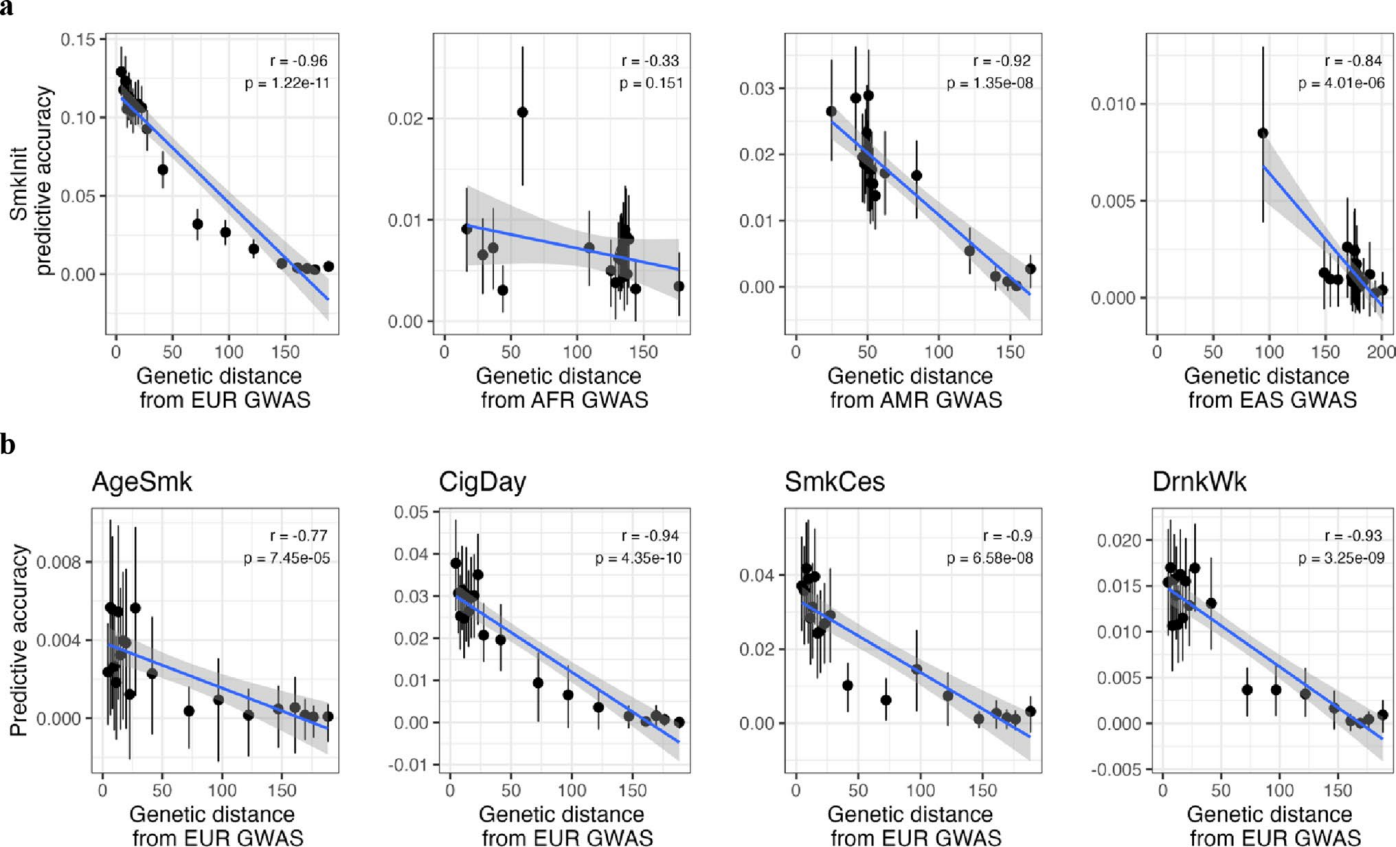
Predictive accuracy of polygenic scores by 20 equally sized bins of genetic distance (GD). **a** All plots refer to the predictive accuracy of SmkInit PRS. The ancestry source of the PRS matches the genetic distance ancestry denoted on the x-axis. Each of the 20 bins, containing *N* = 10,529−10,840 individuals, are plotted by their mean genetic distance so that they are not equally spaced on the x-axis. The blue lines indicate regressions of genetic distance on PRS accuracy with correlations, and their raw *p*-values, reported in each subplot. **b** Shows the predictive accuracy of PRS by EUR GD for each of the remaining four phenotypes: AgeSmk (*N* = 2772−4341 per bin), CigDay (*N* = 2481−4045 per bin), SmkCes (*N* = 3133−4918 per bin), and DrnkWk (*N* = 6263−8527 per bin)

Results for SmkInit are shown in Fig. 3a. Across all discovery GWAS ancestries, there is a decline in the predictive accuracy of the PRS across bins of genetic distance. The correlations between genetic distance and predictive accuracies ranged from *r* = − 0.96 for EUR ancestry to *r* = − 0.33 for AFR ancestry. Figure 3b shows the predictive accuracy of EUR-based PRS across bins of EUR genetic distance for the remaining four phenotypes: AgeSmk, CigDay, SmkCes, and DrnkWk. Correlations for these phenotypes ranged from *r* = − 0.77 to *r* = − 0.94 showing a strong decline in predictive accuracy of EUR-based PRS and genetic distance from the EUR discovery GWAS increases. Full results for all GWAS ancestry/phenotype combinations are included in Supplementary Fig. 3 and Supplementary Table 2.

This pattern holds within ancestry groups as well (Supplementary Fig. 4). For each ancestry we calculated deciles of genetic distance from the EUR discovery GWAS. In 14 of 17 cases (82.4%) the correlation between EUR genetic distance and predictive accuracy of EUR-based scores was negative in sign. For example, within AoU individuals of EUR ancestry, the variance explained by the SmkInit EUR-based PRS was 12.7% in the first decile (i.e., individuals closest to the GWAS SmkInit EUR discovery sample reference point) compared to 8.7% for EUR individuals furthest away (i.e., the tenth decile).

### Reduced Accuracy Explained by MAF, LD, and Heritability Differences

**Fig. 4:**
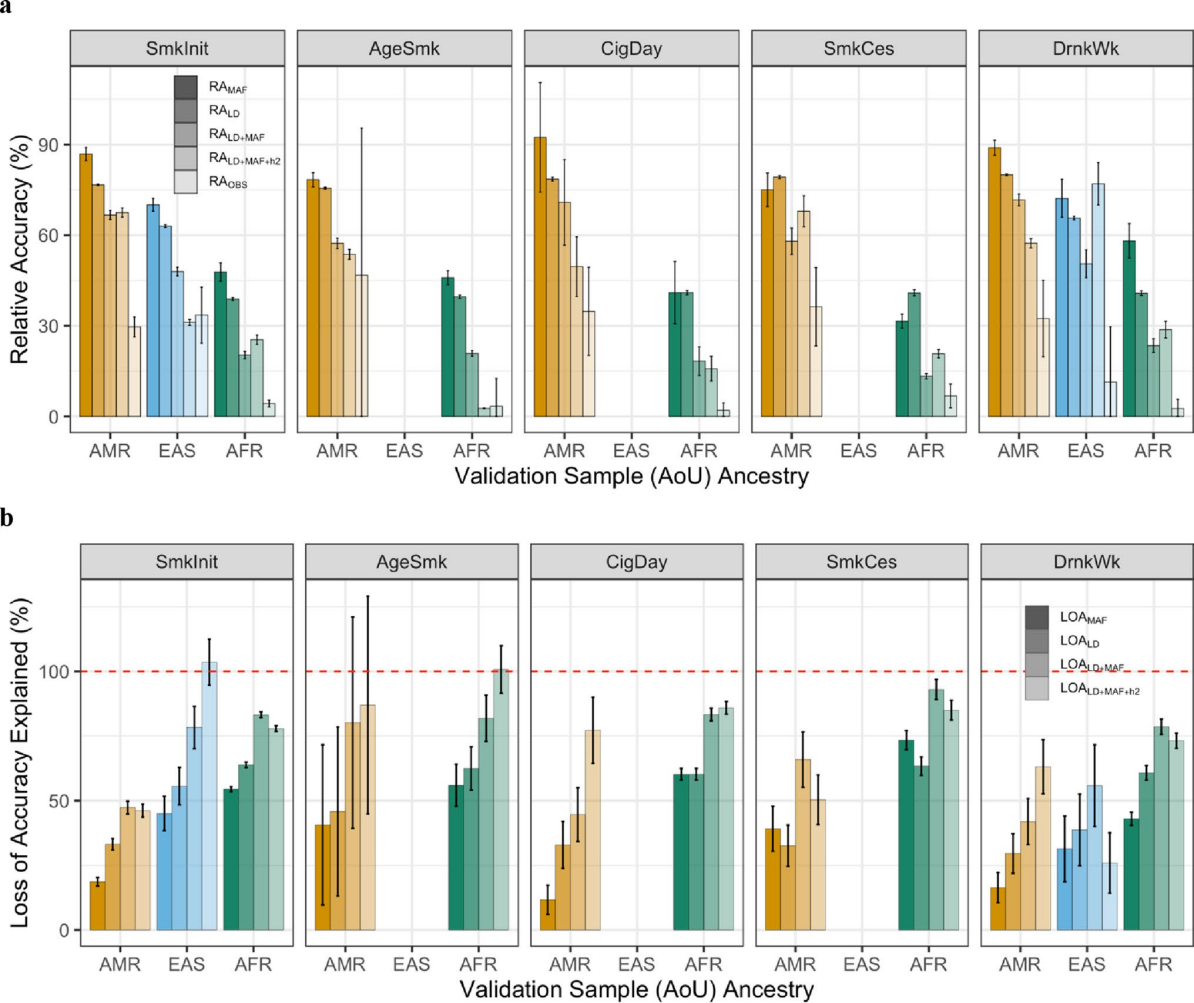
Contribution of genetic architecture differences to reduced EUR-based PRS accuracy across target ancestries and phenotypes. **a** Relative accuracy ($\:RA$) of EUR-based polygenic scores across target validation ancestries (AMR, EAS, AFR) and five smoking and drinking behaviors. Lightest bars represent observed relative accuracy $\:\left({RA}_{OBS}\right),\:$while darker bars represent accuracy predicted by models accounting for differences in linkage disequilibrium (LD), minor allele frequency (MAF), and heritability (h^2^). Predicted $\:RA$ is shown for individual components ($\:{RA}_{MAF}$, $\:{RA}_{LD}$) and their combinations ($\:{RA}_{LD+MAF}\:$and $\:{RA}_{LD+MAF+{h}^{2}}$). **b** Proportion of the loss of PRS accuracy explained ($\:LOA$) by genetic architecture differences, calculated as $\:\left(1-{RA}_{predicted}\right)/\left(1-{RA}_{OBS}\right)\times\:100\%$. Bars illustrate how much of the accuracy gap relative to European samples is attributable to LD, MAF, and/or heritability divergence. All error bars represent 95% confidence intervals derived from normal approximations ($\:\pm\:1.96\times\:SE$)

We evaluated the extent to which population-level differences in allele frequencies (MAF), linkage disequilibrium (LD) patterns, and SNP heritabilities (h^2^) explain the observed decay in PRS transferability using a model derived from population genetic parameters (Methods). Figure 4a displays the observed relative accuracy ($\:{RA}_{OBS})$ alongside accuracies predicted by the full model ($\:{RA}_{LD+MAF+{h}^{2}}$) and more constrained models ($\:{RA}_{LD+MAF},{RA}_{LD}$, and $\:{RA}_{MAF}$). In all cases, the accuracy predicted by MAF and LD alone ($\:{RA}_{LD+MAF}$) was significantly higher than the observed accuracy ($\:{RA}_{OBS}$), confirming that these factors contribute substantially to transferability decline, though they do not fully account for it across all phenotypes. The inclusion of within-ancestry SNP heritability ratios ($\:{RA}_{LD+MAF+{h}^{2}}$) generally resulted in predicted accuracies that were similar to, or slightly lower than, the $\:{RA}_{LD+MAF}$ model, bringing predictions marginally closer to observed values. A notable exception occurred for drinks per week (DrnkWk) in EAS ancestry, where incorporating heritability ratios widened the gap between predicted and observed accuracies.

Mean accuracy prediction varied significantly across ancestry groups. For AMR ancestry, the mean $\:{RA}_{OBS}$ across phenotypes was 36.0%. The $\:{RA}_{LD+MAF}$ model predicted a mean accuracy of 64.9%, which decreased to 59.2% when SNP heritabilities were included. In EAS ancestry, the mean $\:{RA}_{OBS}$ was 22.5%, compared to a predicted 49.2% (54.1% with h^2^ ratios). AFR ancestries showed the lowest transferability, with a mean $\:{RA}_{OBS}$ of only 3.8% and a predicted accuracy of 19.3% (18.7% with h^2^ ratios). Figure 4b quantifies these predicted accuracies into the loss of accuracy (LOA) explained by each model. For example, for SmkInit in EAS ancestries we can explain all of the observed reduced in accuracy across populations through LD, MAF, and heritability differences.

To further disentangle the primary drivers of this reduction, we compared relative accuracy predicted by LD versus MAF alone. Under the assumption that all EUR-based PRS SNPs are causal variants, $\:{RA}_{MAF}\:$estimates were consistently higher than both $\:{RA}_{LD}$ and $\:{RA}_{OBS}$ across nearly all phenotypes and ancestries. This pattern indicates that differences in LD patterns explain a larger proportion of the reduction in EUR-based PRS accuracy than differences in allele frequencies. Figure 4b illustrates that while both factors play a role, the divergence in LD is the predominant component driving the transferability gap. The only observed exception was smoking cessation (SmkCes), where MAF differences appeared to play a more substantial role in reducing accuracy.

### Accuracy by Recombination Rates

**Fig. 5:**
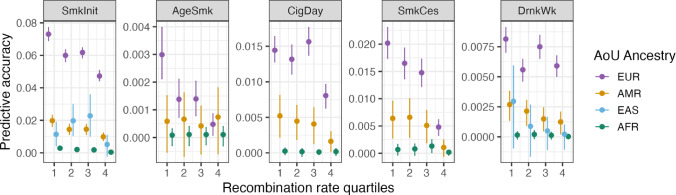
EUR-based PRS predictive accuracy as a function of quartiles of recombination rates (based on 1000 Genomes CEU subsample) for each phenotype. Individuals of EAS ancestry were removed for AgeSmk, CigDay, and SmkCes due to low validation sample sizes. Error bars denote 95% confidence intervals from percentile bootstrapping with 1,000 replications

If differences between populations in LD patterns drive the observed reduction in PRS accuracy, we would expect that SNPs in low recombination regions would transfer better than SNPs in high recombination regions. This is because LD patterns are more similar across populations in genomic regions of low recombination rates. For each phenotype, we binned the EUR-based PRS into quartiles of 1000 Genomes CEU population recombination rates and compared the predictive accuracy of scores within each (Fig. 5). We indeed see that the predictive accuracy of scores tends to decrease as the recombination rates increase for all ancestries, even within EUR ancestries in which the discovery GWAS and validation sample ancestry is matched. The predictive accuracy of scores declines, on average, 53%, 48%, 46%, and 74% in EUR, AFR, AMR, and EAS ancestries, respectively, from the 1st to the 4th quartiles of recombination rates. The relative accuracy of scores across phenotypes also tend to decline across quartiles of recombination rates: from the 1st to 4th quartiles the relative accuracy of EUR-based scores drops 34% in AMR ancestries, 30% in AFR ancestries, and 72% in EAS ancestries (we note EAS ancestries are only included for two phenotypes). This further suggests that differences in LD explain a sizable portion of the observed reduction in accuracy. However, even in the lowest recombination quartile we still observe a substantial reduction in PRS accuracy across ancestries. Averaging across phenotypes, the accuracy of scores relative to EUR ancestry in the lowest recombination quartile was 32%, 2.7%, and 26% for AMR, AFR, and EAS ancestries, respectively, indicating that LD differences do not account for all of the observed decline in accuracy. We repeated all analyses using recombination rates from an admixed African ancestry sample finding very similar patterns of results (Supplementary Fig. 5).

## Discussion

Polygenic risk scores (PRS) have emerged as potential tools in predicting individual health outcomes based on genomic data (Lewis and Vassos 2022; Murray et al. 2021; Yanes et al. 2020). Their utility across diverse populations, however, has been limited by the overrepresentation of European (EUR) ancestries in genome-wide association discovery samples. The present study evaluated the cross-population transferability of polygenic scores for substance use, moving beyond simple observations of performance decay to a mechanistic decomposition of its drivers. By utilizing a theoretical framework derived from Wang et al. (2020), we quantified the relative contributions of linkage disequilibrium (LD), minor allele frequency (MAF), and SNP-based heritability (h^2^) to the loss of predictive accuracy.

We found that the mean observed relative accuracy of European-based PRS was 36%, 22.5%, and 3.8% in AMR, EAS, and AFR ancestries, respectively. These values are approximately half of those found for anthropometric and physical diseases. Martin et al. (2019) report relative accuracies of ~ 60% in AMR ancestries, ~ 50% in EAS ancestries, and ~ 20% in AFR ancestries. Similarly, Wang et al. (2020) report relative accuracies of 64% in EAS ancestries and 24% in AFR ancestries (AMR ancestries were not included in their analyses). It may be that for behavioral traits, like substance use behaviors studied here, cultural and environmental factors not only reduce the overall variance explained by polygenic scores, but also the transferability of such scores across populations (Kamiza et al. 2022; Mostafavi et al. 2020).

While these group-level comparisons can be helpful in describing broad patterns, they may mask more nuanced relationships of PRS transferability. Further, genetic ancestry classification is reliant on subjective thresholds that often result in removal of individuals who are not assigned to a single ancestry group (Lewis et al. 2022). To avoid these pitfalls, we evaluated the transferability of scores across genetic distance from the discovery GWAS sample. We found that for all phenotypes the predictive accuracy of EUR-based scores declines across bins of genetic ancestry distance from the EUR discovery GWAS. This reduction in accuracy by increasing genetic distance held even within traditionally defined genetic ancestry groups. For example, within individuals of EUR ancestry, those closer in genetic distance to the GWAS discovery sample had higher PRS accuracy than those farther away. The continuous decline in accuracy as individuals move further from the discovery GWAS is consistent with prior work focusing on height (Ding et al. 2023), suggesting a pervasive relationship across traits and samples.

The observed reductions in predictive accuracy of scores were found to be partially explained by differences across populations in linkage disequilibrium (LD), minor allele frequencies (MAF), and SNP heritabilites. Our results suggested that, together, differences in MAF and LD explained, on average, 56.0%, 67.1%, and 84.0% of the loss of predictive accuracy in AMR, EAS, and AFR ancestries, respectively. These estimates are very similar to recent work based on anthropometric and medical phenotypes (Wang et al. 2020). In other words, while polygenic scores for substance use behaviors may show comparatively lower variance explained and a greater reduction in transferability across populations, as compared to anthropometric and disease outcomes, the extent to which factors like LD and allele frequency differences account for this decline are very similar. Further accounting for differences across populations in SNP heritability estimates accounted for only a small proportion of the loss of accuracy for most ancestry, phenotype combinations. Notably, accounting for SNP heritability only minimally improved these estimates, with one exception: drinks per week (DrnkWk) in EAS ancestries. In this case, the SNP heritability ($$\:{h}_{EAS}^{2}=0.06$$$, excluding chromosome 12 which contains *ALDH2*) was higher than in EUR ancestries ($$\:{h}_{EUR}^{2}=0.04$$), reducing the proportion of explained accuracy loss.

However, after accounting for differences in MAF, LD, and SNP heritabilities, substantial reductions in accuracy remain across populations and phenotypes. This suggests that other factors, such as differences in causal variant effect sizes or interactions between genes and environmental factors, are likely contributing to the remaining decline in PRS accuracy. Indeed, recent work has started to explore causal the impacts of effect size heterogeneity across populations finding somewhat inconsistent results (Hou et al. 2023; Patel et al. 2022). Future work on the influence of these factors will be important for the development of equally accurate polygenic scores regardless of genetic ancestry (Mostafavi et al. 2020; Wojcik et al. 2019).

The current study should be interpreted in the context of several limitations. First, the model used to estimate the contributions of LD and MAF differences to transferability of scores relies on several untestable assumptions (Wang et al. 2020). In practice we do not know which variants are causal or the true heritabilities in each population. We use the same heuristic to define candidate causal variants as the authors of the method which worked well in simulations, but we cannot know how well this identifies true causal variants.

Second, despite efforts to include discovery GWAS and target samples of greater diversity in genetic ancestries, we still are reliant on samples that are predominantly European populations. For example, EUR ancestry GWAS sample sizes for smoking initiation were ~ 22x larger than for AFR ancestry, and ~9x larger than for AMR and EAS ancestries. Similarly, the EUR stratified target sample was ~ 22x larger than the EAS sample, ~3x larger than the AMR sample, and ~ 2.5x larger than the AFR sample. The lack of diversity in discovery GWAS samples means that polygenic scores will continue to underperform in non-European populations unless more effort is made to include diverse populations in the initial GWAS research. The lack of diversity in target samples hinders our ability to understand the patterns of predictive accuracy and transferability across all individuals. Recent initiatives like the All of Us research program (Bick et al. 2024) are steps in the right direction, but more work is needed to ensure that all populations benefit equally from advances in genomic research.

An additional limitation of the current work is its focus on ancestry-stratified polygenic scores. We did not incorporate trans-ancestry PRS which may better account for the continuous nature of genetic ancestry. Development of trans-ancestry polygenic scoring methods is an active area of research (Cavazos and Witte 2021; Ge et al. 2022; Hoggart et al. 2023). We look forward to extending this work using such scores in the future. Finally, we focus exclusively on tobacco use and alcohol consumption in the current work given the large and ancestrally diverse discovery GWAS samples available. Future work incorporating other behavioral traits and psychiatric diseases will add important information on how patterns of prediction and transferability across populations align with, or differ from, what has been found for physical health and disease outcomes. Improving the accuracy and transferability of PRS will require not only more diverse GWAS samples but also a deeper understanding of how genetic and environmental factors interact to shape complex behaviors. Until these challenges are addressed, the utility of polygenic scores will remain limited.

## Supplementary Information

Below is the link to the electronic supplementary material.

Supplementary Material 1.

Supplementary Material 2.

## Data Availability

Genomic summary data from the GSCAN consortium used in the present work is publicly available (https://doi.org/10.13020/przg-dp88).

## References

[CR2] Bick AG, Metcalf GA, Mayo KR, Lichtenstein L, Rura S, Carroll RJ, Musick A, Linder JE, Jordan IK, Nagar SD, Sharma S, Meller R, Basford M, Boerwinkle E, Cicek MS, Doheny KF, Eichler EE, Gabriel S, Gibbs RA, NIH All of Us Research Program Staff (2024) Genomic data in the All of Us Research Program. Nature 1–7. 10.1038/s41586-023-06957-x 10.1038/s41586-023-06957-x PMC10937371 38374255

[CR3] Bitarello BD, Mathieson I (2020) Polygenic Scores for Height in Admixed Populations. G3 Genes|Genomes|Genetics 10(11):4027–4036. 10.1534/g3.120.401658 32878958 10.1534/g3.120.401658 PMC7642950

[CR4] Cavazos TB, Witte JS (2021) Inclusion of variants discovered from diverse populations improves polygenic risk score transferability. Hum Genet Genomics Adv 2(1):100017. 10.1016/j.xhgg.2020.100017 10.1016/j.xhgg.2020.100017 PMC7869832 33564748

[CR5] Chang CC, Chow CC, Tellier LC, Vattikuti S, Purcell SM, Lee JJ (2015) Second-generation PLINK: Rising to the challenge of larger and richer datasets. GigaScience 4:7. 10.1186/s13742-015-0047-8 25722852 10.1186/s13742-015-0047-8 PMC4342193

[CR6] Dey R, Lee S (2019) Asymptotic properties of Principal Component Analysis and shrinkage-bias adjustment under the Generalized Spiked Population model. J Multivar Anal 173:145–164. 10.1016/j.jmva.2019.02.007 32831421 10.1016/j.jmva.2019.02.007 PMC7441582

[CR7] Ding Y, Hou K, Xu Z, Pimplaskar A, Petter E, Boulier K, Privé F, Vilhjálmsson BJ, Loohuis O, L. M., Pasaniuc B (2023) Polygenic scoring accuracy varies across the genetic ancestry continuum. Nature 618(7966):7966. 10.1038/s41586-023-06079-4 10.1038/s41586-023-06079-4 PMC10284707 37198491

[CR8] Duncan L, Shen H, Gelaye B, Meijsen J, Ressler K, Feldman M, Peterson R, Domingue B (2019) Analysis of polygenic risk score usage and performance in diverse human populations. Nat Commun 10(1). 10.1038/s41467-019-11112-0 10.1038/s41467-019-11112-0 PMC6658471 31346163

[CR9] Ge T, Irvin MR, Patki A, Srinivasasainagendra V, Lin Y-F, Tiwari HK, Armstrong ND, Benoit B, Chen C-Y, Choi KW, Cimino JJ, Davis BH, Dikilitas O, Etheridge B, Feng Y-CA, Gainer V, Huang H, Jarvik GP, Kachulis C, Karlson EW (2022) Development and validation of a trans-ancestry polygenic risk score for type 2 diabetes in diverse populations. Genome Med 14(1):70. 10.1186/s13073-022-01074-2 35765100 10.1186/s13073-022-01074-2 PMC9241245

[CR1] Genomes Project Consortium, Auton A, Brooks LD, Durbin RM, Garrison EP, Kang HM, Korbel JO, Marchini JL, McCarthy S, McVean GA, Abecasis GR (2015) A global reference for human genetic variation. Nature 526(7571):68–74. 10.1038/nature15393 26432245 10.1038/nature15393 PMC4750478

[CR10] Hinch AG, Tandon A, Patterson N, Song Y, Rohland N, Palmer CD, Chen GK, Wang K, Buxbaum SG, Akylbekova EL, Aldrich MC, Ambrosone CB, Amos C, Bandera EV, Berndt SI, Bernstein L, Blot WJ, Bock CH, Boerwinkle E, Myers SR (2011) The landscape of recombination in African Americans. Nature 476(7359):170–175. 10.1038/nature10336 21775986 10.1038/nature10336 PMC3154982

[CR11] Hoggart C, Choi SW, García-González J, Souaiaia T, Preuss M, O’Reilly P (2023) BridgePRS: a powerful trans-ancestry Polygenic Risk Score method. bioRxiv 20230217528938. 10.1101/2023.02.17.528938

[CR12] Hou K, Ding Y, Xu Z, Wu Y, Bhattacharya A, Mester R, Belbin GM, Buyske S, Conti DV, Darst BF, Fornage M, Gignoux C, Guo X, Haiman C, Kenny EE, Kim M, Kooperberg C, Lange L, Manichaikul A, Pasaniuc B (2023) Causal effects on complex traits are similar for common variants across segments of different continental ancestries within admixed individuals. Nat Genet 55(4): Article 4. 10.1038/s41588-023-01338-6 10.1038/s41588-023-01338-6 PMC11120833 36941441

[CR13] Kachuri L, Chatterjee N, Hirbo J, Schaid DJ, Martin I, Kullo IJ, Kenny EE, Pasaniuc B, Witte JS, Ge T (2024) Principles and methods for transferring polygenic risk scores across global populations. Nat Rev Genet 25(1):8–25. 10.1038/s41576-023-00637-2 37620596 10.1038/s41576-023-00637-2 PMC10961971

[CR14] Kamiza AB, Toure SM, Vujkovic M, Machipisa T, Soremekun OS, Kintu C, Corpas M, Pirie F, Young E, Gill D, Sandhu MS, Kaleebu P, Nyirenda M, Motala AA, Chikowore T, Fatumo S (2022) Transferability of genetic risk scores in African populations. Nat Med 28(6):1163–1166. 10.1038/s41591-022-01835-x 35654908 10.1038/s41591-022-01835-x PMC9205766

[CR15] Lambert SA, Abraham G, Inouye M (2019) Towards clinical utility of polygenic risk scores. Hum Mol Genet 28(R2):R133–R142. 10.1093/hmg/ddz187 31363735 10.1093/hmg/ddz187

[CR16] Lee SH, Goddard ME, Wray NR, Visscher PM (2012) A better coefficient of determination for genetic profile analysis. Genet Epidemiol 36(3):214–224. 10.1002/gepi.21614 22714935 10.1002/gepi.21614

[CR18] Lewis CM, Vassos E (2022) Polygenic Scores in Psychiatry: On the Road From Discovery to Implementation. Am J Psychiatry 179(11):800–806. 10.1176/appi.ajp.20220795 36317334 10.1176/appi.ajp.20220795

[CR17] Lewis ACF, Molina SJ, Appelbaum PS, Dauda B, Di Rienzo A, Fuentes A, Fullerton SM, Garrison NA, Ghosh N, Hammonds EM, Jones DS, Kenny EE, Kraft P, Lee SS-J, Mauro M, Novembre J, Panofsky A, Sohail M, Neale BM, Allen DS (2022) Getting genetic ancestry right for science and society. Science 376(6590):250–252. 10.1126/science.abm7530 35420968 10.1126/science.abm7530 PMC10135340

[CR19] Martin AR, Kanai M, Kamatani Y, Okada Y, Neale BM, Daly MJ (2019) Clinical use of current polygenic risk scores may exacerbate health disparities. Nat Genet 51(4). 10.1038/s41588-019-0379-x 10.1038/s41588-019-0379-x PMC6563838 30926966

[CR20] Mostafavi H, Harpak A, Agarwal I, Conley D, Pritchard JK, Przeworski M (2020) Variable prediction accuracy of polygenic scores within an ancestry group. eLife 9:e48376. 10.7554/eLife.48376 31999256 10.7554/eLife.48376 PMC7067566

[CR21] Murray GK, Lin T, Austin J, McGrath JJ, Hickie IB, Wray NR (2021) Could Polygenic Risk Scores Be Useful in Psychiatry? A Review. JAMA Psychiatry 78(2):210–219. 10.1001/jamapsychiatry.2020.3042 33052393 10.1001/jamapsychiatry.2020.3042

[CR22] Novembre J, Barton NH (2018) Tread Lightly Interpreting Polygenic Tests of Selection. Genetics 208(4):1351–1355. 10.1534/genetics.118.300786 29618592 10.1534/genetics.118.300786 PMC5886544

[CR23] Patel RA, Musharoff SA, Spence JP, Pimentel H, Tcheandjieu C, Mostafavi H, Sinnott-Armstrong N, Clarke SL, Smith CJ, Durda PP, Taylor KD, Tracy R, Liu Y, Johnson WC, Aguet F, Ardlie KG, Gabriel S, Smith J, Nickerson DA, Pritchard JK (2022) Genetic interactions drive heterogeneity in causal variant effect sizes for gene expression and complex traits. Am J Hum Genet 109(7):1286–1297. 10.1016/j.ajhg.2022.05.014 35716666 10.1016/j.ajhg.2022.05.014 PMC9300878

[CR24] Saunders GRB, Wang X, Chen F, Jang S-K, Liu M, Wang C, Gao S, Jiang Y, Khunsriraksakul C, Otto JM, Addison C, Akiyama M, Albert CM, Aliev F, Alonso A, Arnett DK, Ashley-Koch AE, Ashrani AA, Barnes KC, Vrieze S (2022) Genetic diversity fuels gene discovery for tobacco and alcohol use. Nature 612(7941): Article 7941. 10.1038/s41586-022-05477-4 10.1038/s41586-022-05477-4 PMC9771818 36477530

[CR25] Sollis E, Mosaku A, Abid A, Buniello A, Cerezo M, Gil L, Groza T, Güneş O, Hall P, Hayhurst J, Ibrahim A, Ji Y, John S, Lewis E, MacArthur JAL, McMahon A, Osumi-Sutherland D, Panoutsopoulou K, Pendlington Z, Harris LW (2023) The NHGRI-EBI GWAS Catalog: Knowledgebase and deposition resource. Nucleic Acids Res 51(D1):D977–D985. 10.1093/nar/gkac1010 36350656 10.1093/nar/gkac1010 PMC9825413

[CR26] Torkamani A, Wineinger NE, Topol EJ (2018) The personal and clinical utility of polygenic risk scores. Nat Rev Genet 19(9): Article 9. 10.1038/s41576-018-0018-x 10.1038/s41576-018-0018-x 29789686

[CR27] Wang Y, Guo J, Ni G, Yang J, Visscher PM, Yengo L (2020) Theoretical and empirical quantification of the accuracy of polygenic scores in ancestry divergent populations. Nat Commun 11(1). 10.1038/s41467-020-17719-y 10.1038/s41467-020-17719-y PMC7395791 32737319

[CR28] Wojcik GL, Graff M, Nishimura KK, Tao R, Haessler J, Gignoux CR, Highland HM, Patel YM, Sorokin EP, Avery CL, Belbin GM, Bien SA, Cheng I, Cullina S, Hodonsky CJ, Hu Y, Huckins LM, Jeff J, Justice AE, Carlson CS (2019) Genetic analyses of diverse populations improves discovery for complex traits. Nature 570(7762):514–518. 10.1038/s41586-019-1310-4 31217584 10.1038/s41586-019-1310-4 PMC6785182

[CR29] Yanes T, McInerney-Leo AM, Law MH, Cummings S (2020) The emerging field of polygenic risk scores and perspective for use in clinical care. Hum Mol Genet 29(R2):R165–R176. 10.1093/hmg/ddaa136 32620971 10.1093/hmg/ddaa136

[CR30] Zhang D, Dey R, Lee S (2020) Fast and robust ancestry prediction using principal component analysis. Bioinformatics 36(11):3439–3446. 10.1093/bioinformatics/btaa152 32196066 10.1093/bioinformatics/btaa152 PMC7267814

