# Supplementary material for: Insights into the Cross-Population Transferability of Polygenic Scores for Substance Use
Source: Behav Genet. 2026 Apr 29;56(3):119–30. doi: 10.1007/s10519-026-10265-1 (PMC13201358; doi:10.1007/s10519-026-10265-1)

**Supplementary Figure 1.** Principal components (PC) space of All of Us sample projected onto 1000 Genomes for the first five dimensions. Colored points are individuals in All of Us colored by ancestry category; black points are individuals from 1000 Genomes.


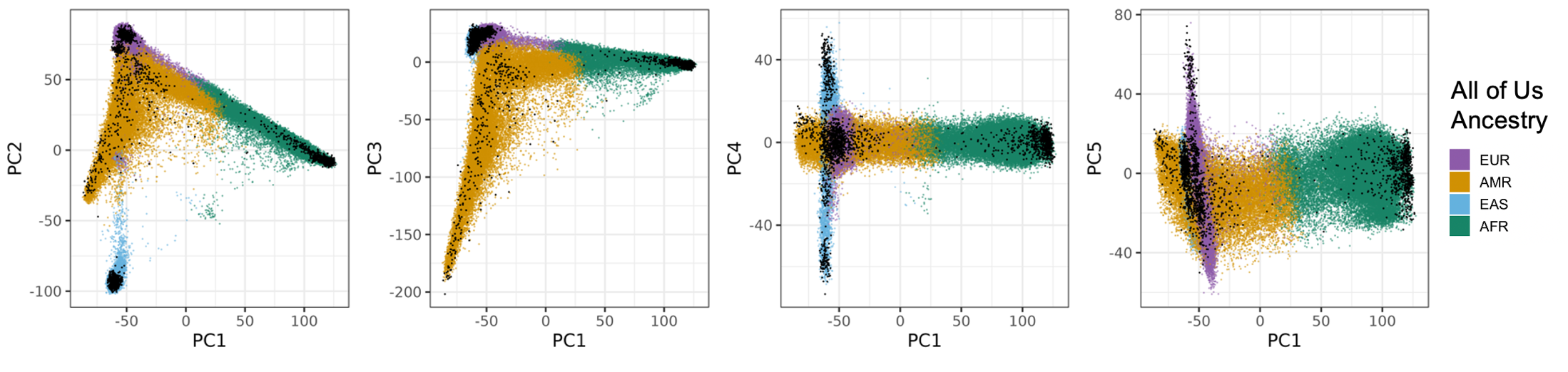


**Supplementary Figure 2.** Mapping of ancestry-stratified GWAS discovery samples (from Saunders et al., 2022) onto PC space of the All of Us validation sample. Sequence shows how we used the 1000 Genomes per ancestry multidimensional scaling (MDS) points and 1000 Genomes per ancestry PC centroids to map the GWAS discovery sample MDS points onto the All of Us PC space. Row 1 shows the MDS components for the GWAS discovery samples per ancestry (colored squares) and 1000 Genomes per ancestry (black dots). Row 2 shows the results from a Procrustes transformation of the 1000 Genomes MDS points (black dots) onto the PC space of the All of Us samples (colored dots). Row 3 shows the output from the Procrustes transformation applied to the GWAS discovery sample MDS points (black squares) onto the PC space of the All of Us sample (colored dots). The end result in row 3 is a PC space based on All of Us individuals that also contains discovery GWAS per-ancestry MDS components while retaining the relative similarities.


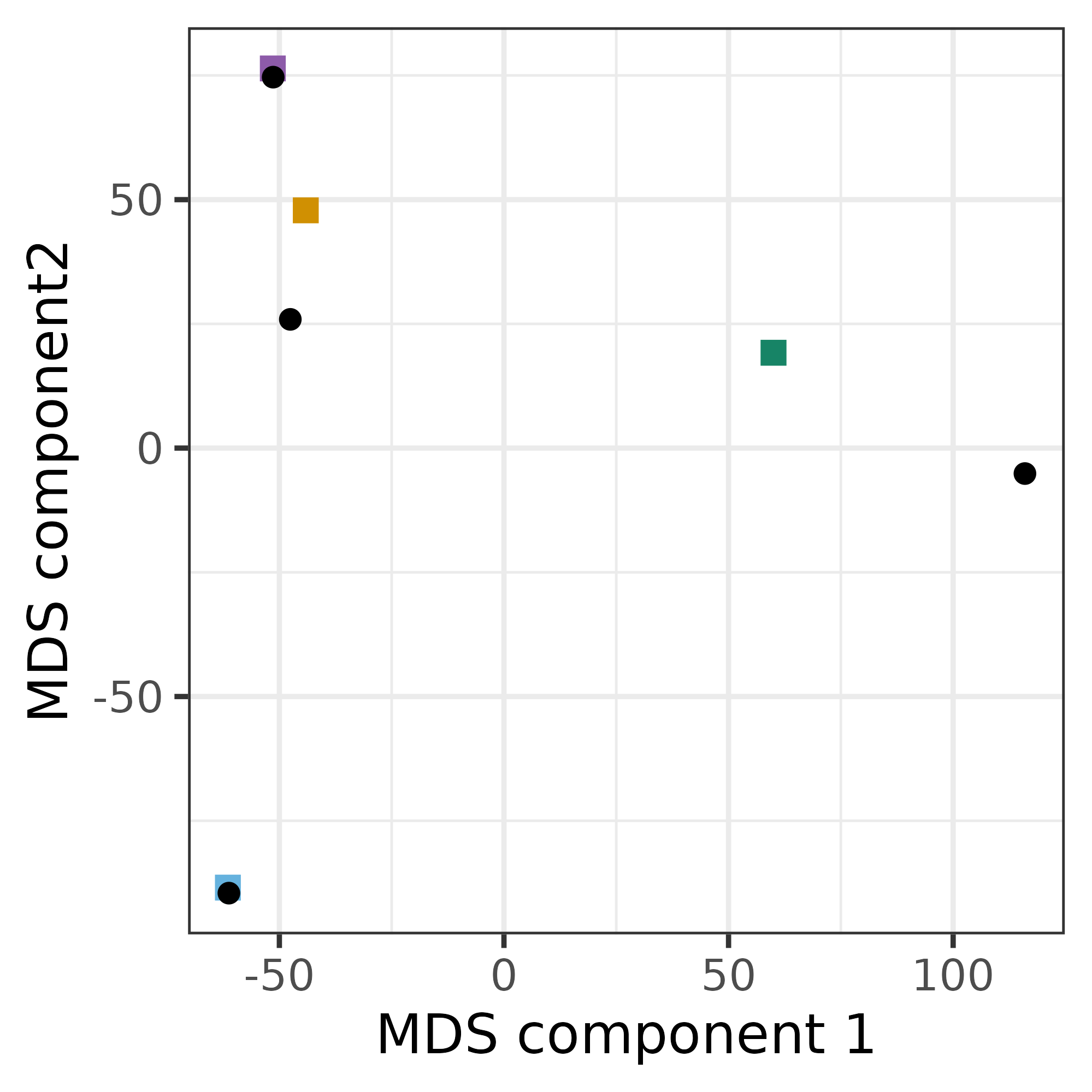

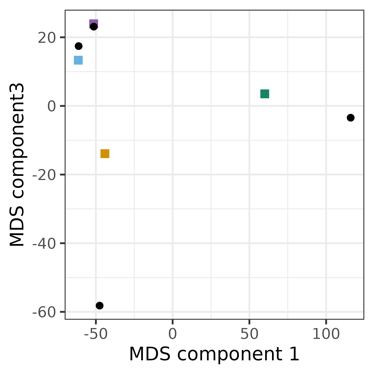

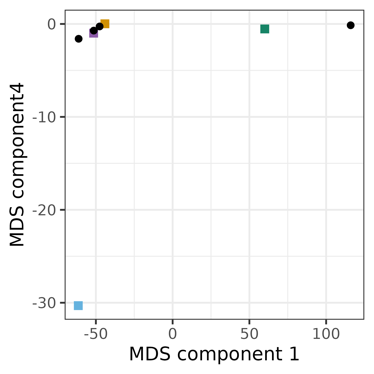

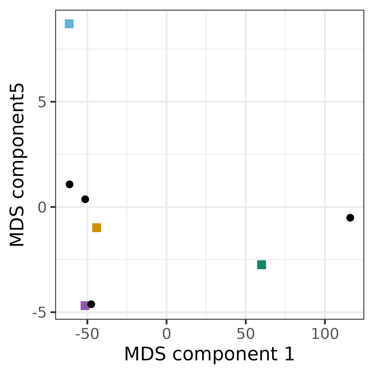


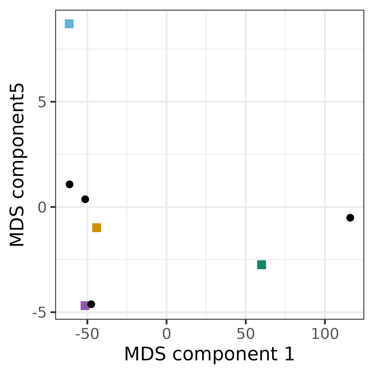

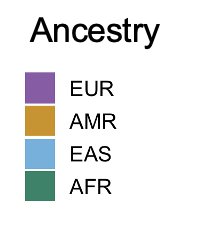


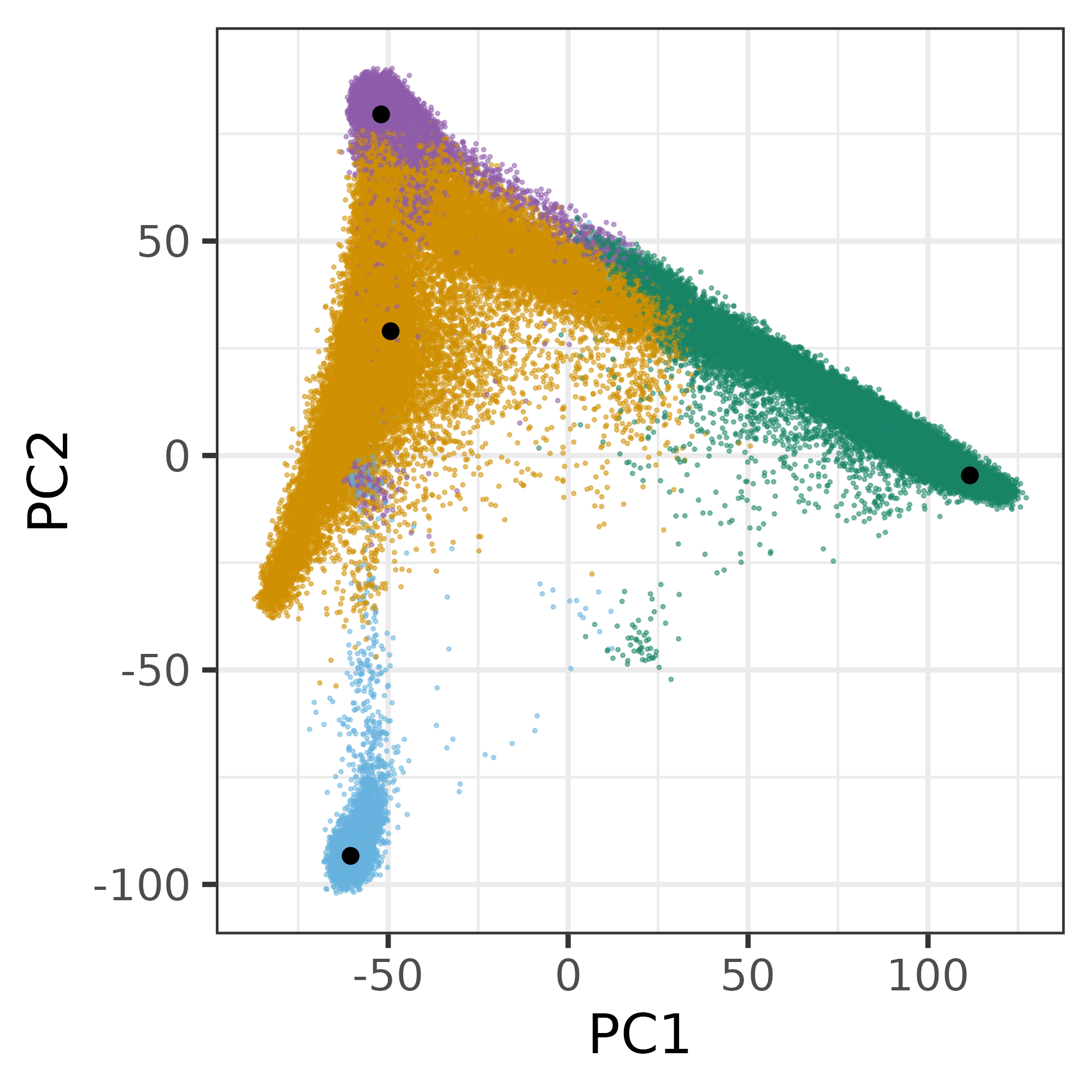

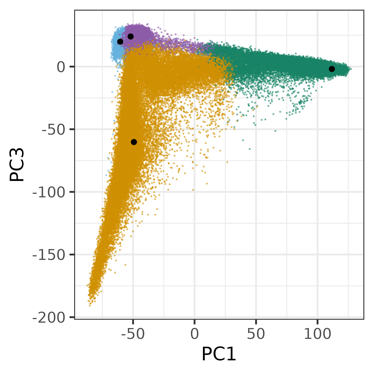

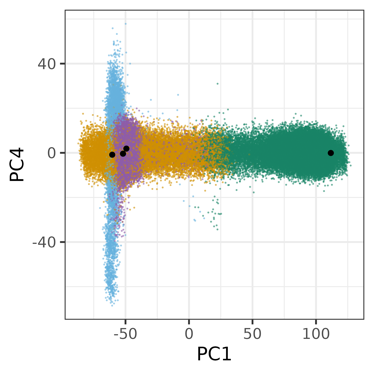

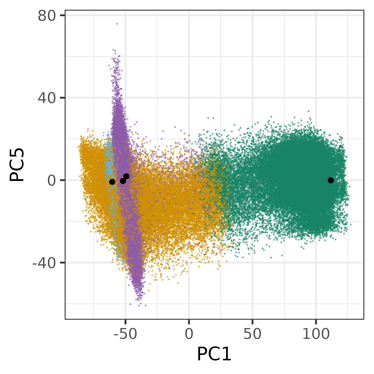


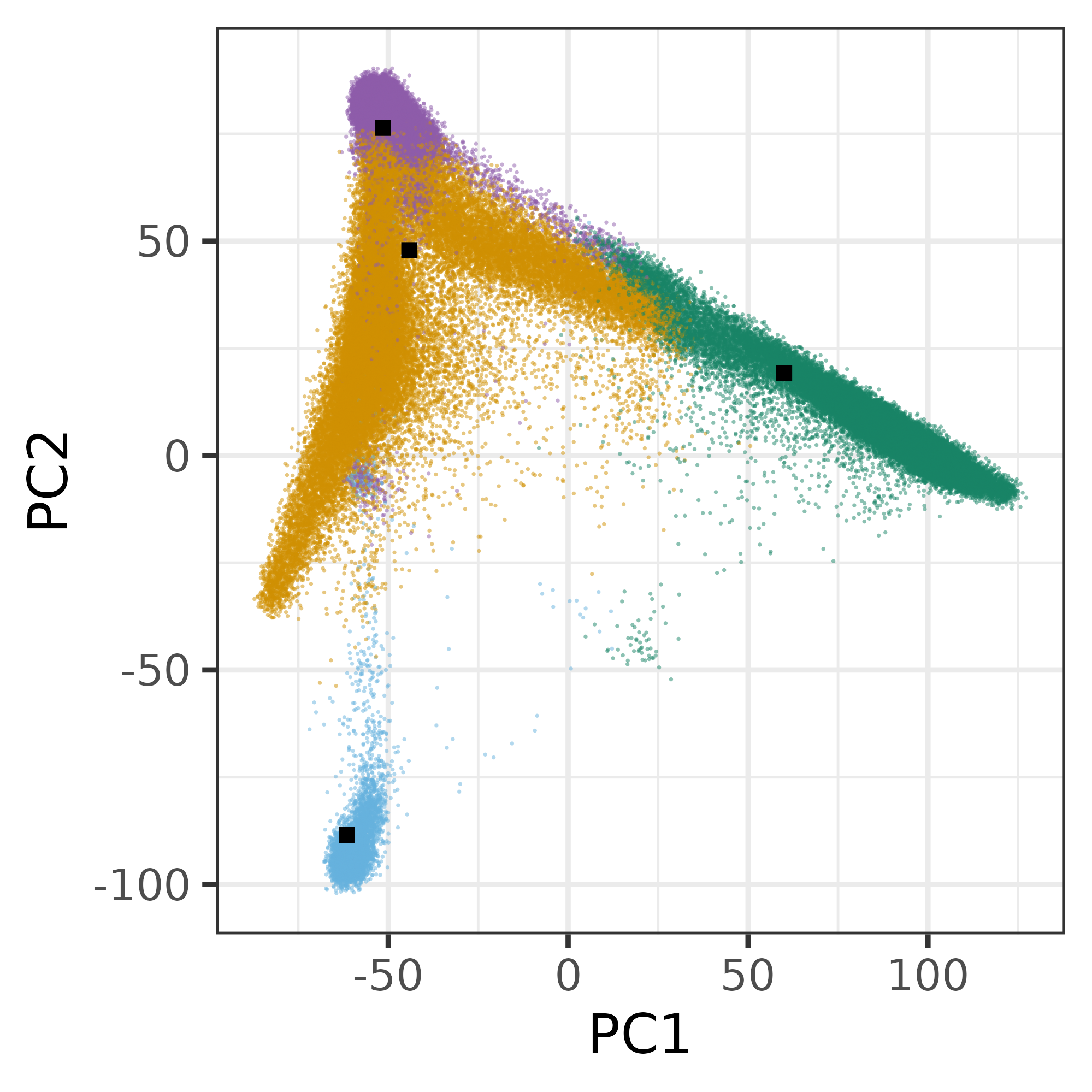

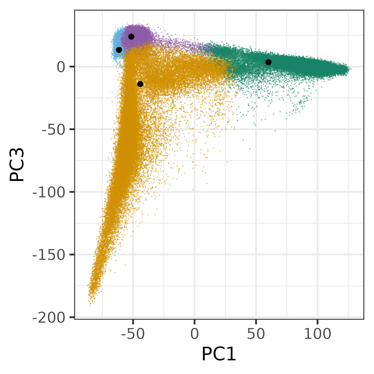

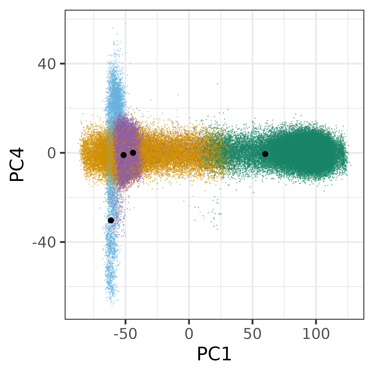

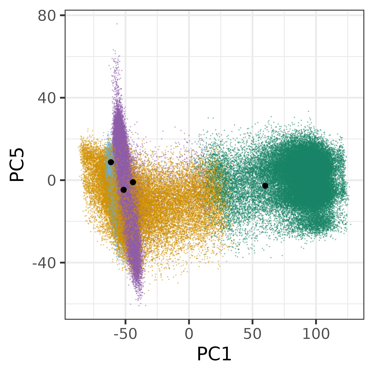


**Supplementary Figure 3.** Predictive accuracy of PRS by 20 equally sized bins of genetic distance (GD) for every phenotype/GD ancestry combination (except for SmkInit which is displayed in Main Figure 3). The ancestry source of the PRS matches the genetic distance ancestry denoted on the x-axis. Each of the 20 bins are plotted by their mean genetic distance so that they are not equally spaced on the x-axis. The blue lines indicate regressions of genetic distance on PRS accuracy with correlations, and their raw *p*-values, reported in each subplot.


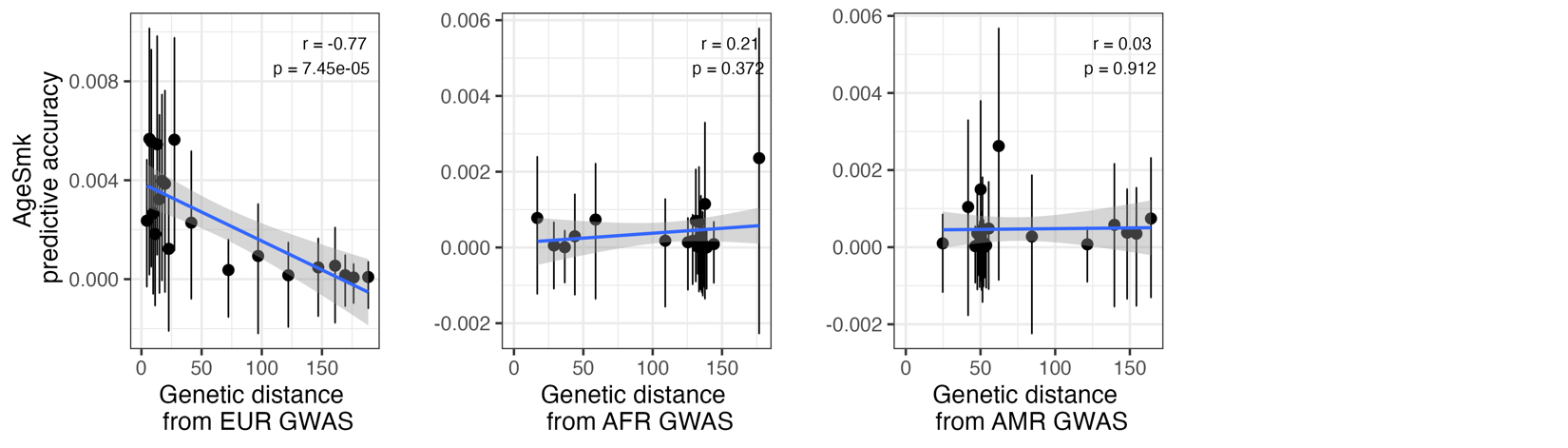


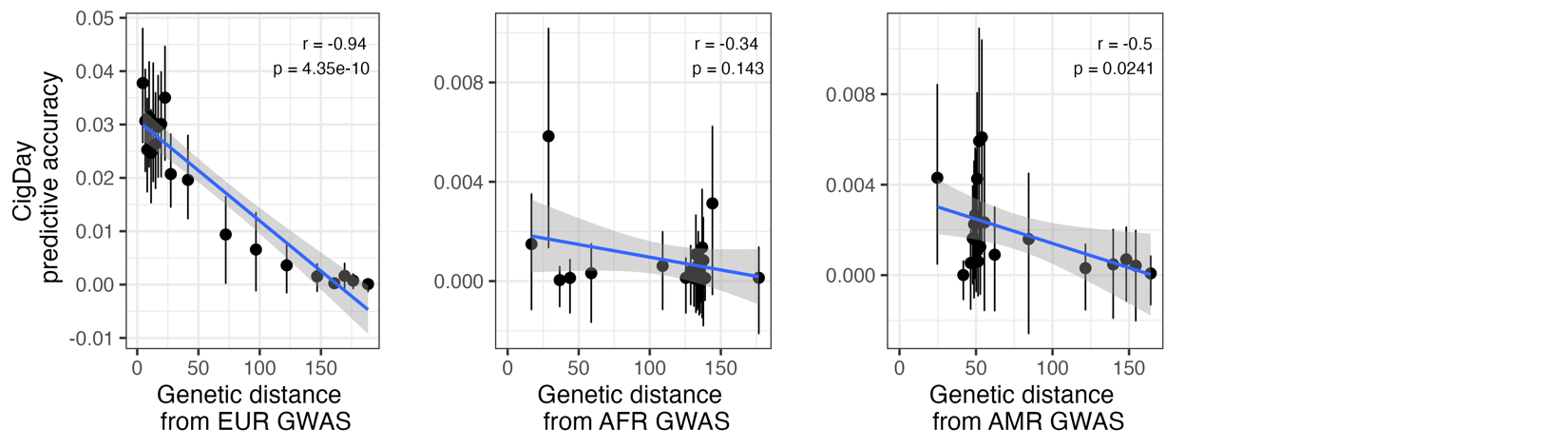


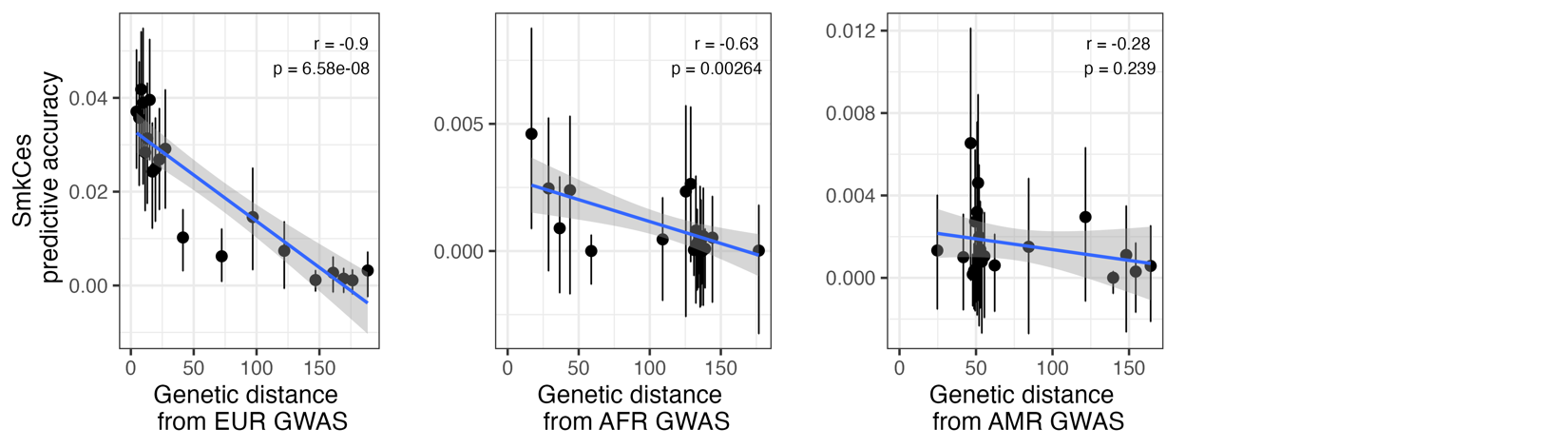


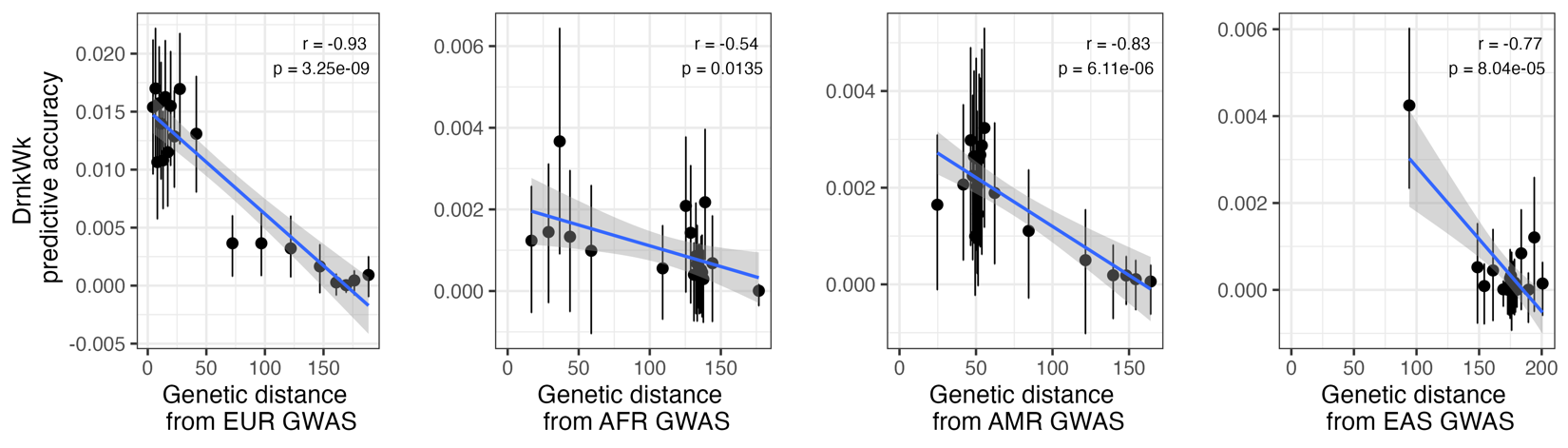


**Supplementary Figure 4**. Predictive accuracy of PRS by 10 equally sized bins within All of Us ancestry of EUR genetic distance for each phenotype. Each of the 10 bins per ancestry are plotted by their mean EUR genetic distance so that they are not equally spaced on the x-axis. The blue lines indicate regressions of genetic distance on PRS accuracy with correlations, and their raw *p*-values, reported in each subplot.


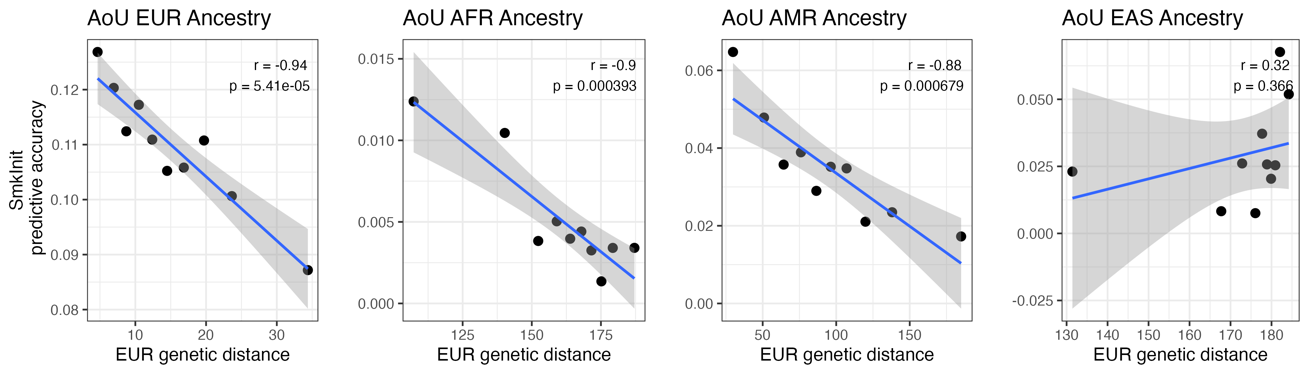


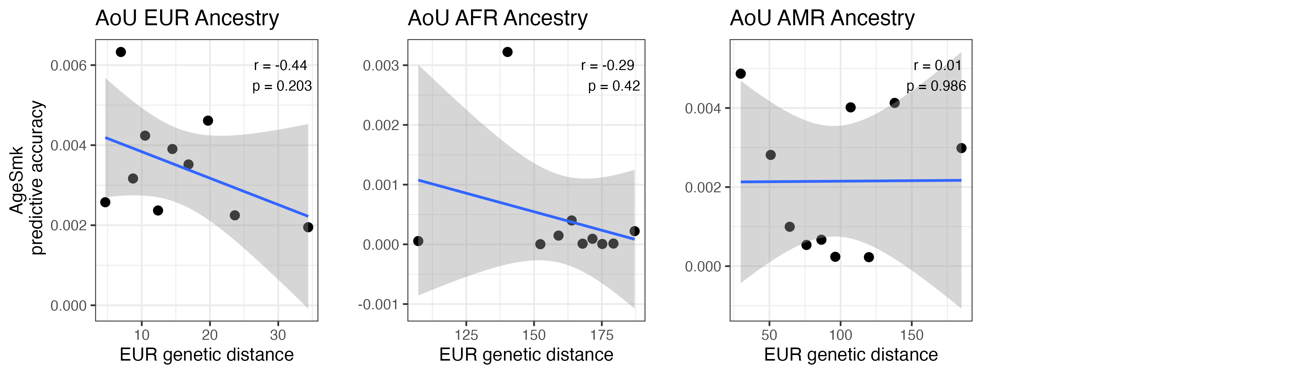


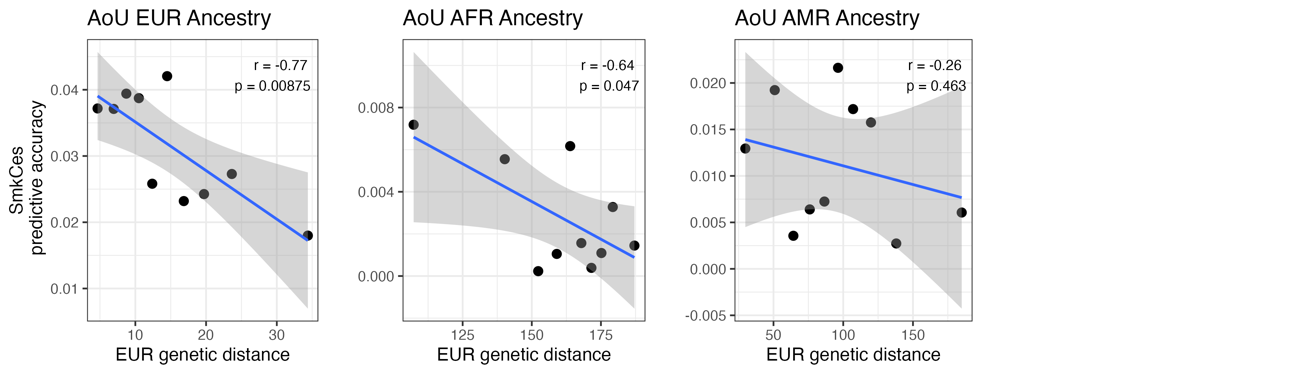


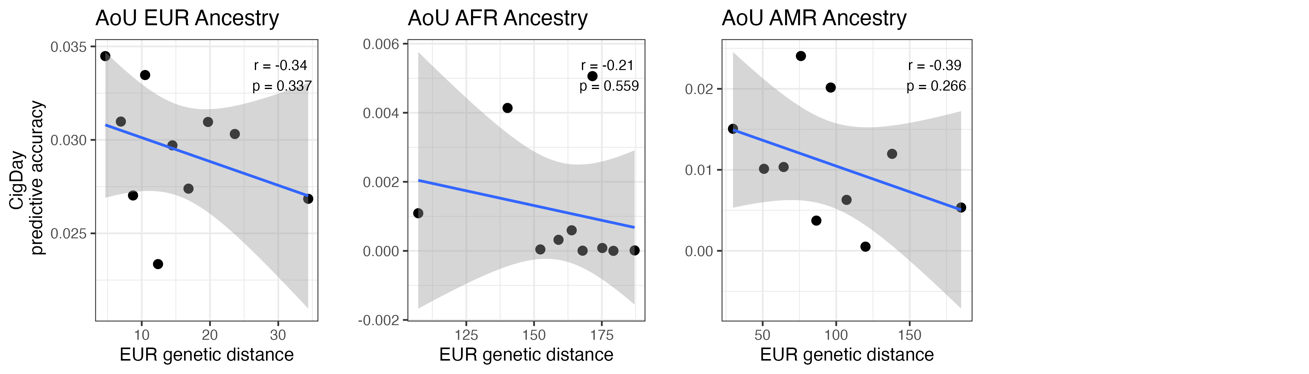


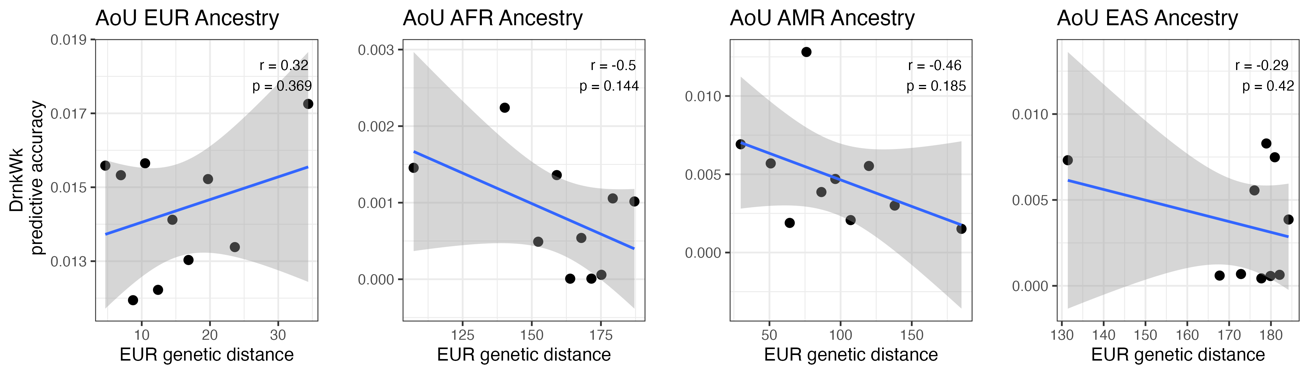


**Supplementary Figure 5.** EUR-based PRS predictive accuracy as a function of quartiles of recombination rates (based on an African American sample [Hinch et al.]) for each phenotype. Individuals of EAS ancestry were removed for AgeSmk, CigDay, and SmkCes due to low validation sample sizes. Error bars denote 95% confidence intervals from percentile bootstrapping with 1,000 replications.


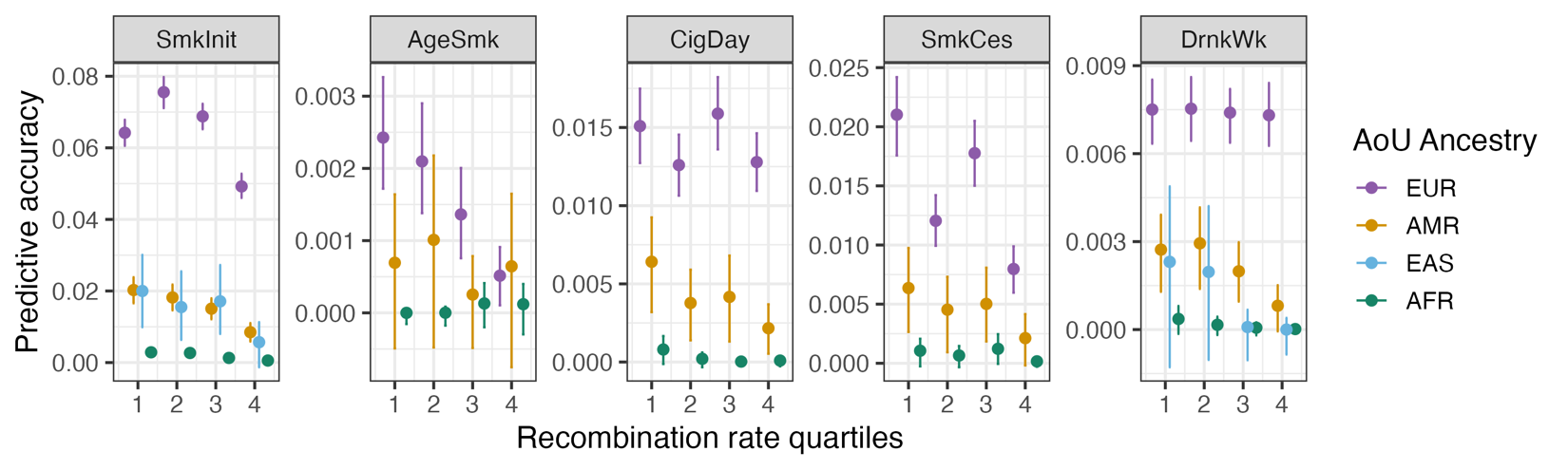

Supplement: Supplementary file 2 — Supplementary Material 2. [file 10519_2026_10265_MOESM2_ESM.docx]
